# Zinc and Cadmium in the Aetiology and Pathogenesis of Osteoarthritis and Rheumatoid Arthritis

**DOI:** 10.3390/nu13010053

**Published:** 2020-12-26

**Authors:** Theoharris Frangos, Wolfgang Maret

**Affiliations:** Metal Metabolism Group, Department of Nutritional Sciences, School of Life Course Sciences, Faculty of Life Sciences and Medicine, King’s College London, London SE1 9NH, UK; theoharris.frangos@kcl.ac.uk

**Keywords:** osteoarthritis, rheumatoid arthritis, zinc, cadmium

## Abstract

Osteoarthritis (OA) and rheumatoid arthritis (RA) are inflammatory articular conditions with different aetiology, but both result in joint damage. The nutritionally essential metal zinc (Zn^2+^) and the non-essential metal cadmium (Cd^2+^) have roles in these arthritic diseases as effectors of the immune system, inflammation, and metabolism. Despite both metal ions being redox-inert in biology, they affect the redox balance. It has been known for decades that zinc decreases in the blood of RA patients. It is largely unknown, however, whether this change is only a manifestation of an acute phase response in inflammation or relates to altered availability of zinc in tissues and consequently requires changes of zinc in the diet. As a cofactor in over 3000 human proteins and as a signaling ion, zinc affects many pathways relevant for arthritic disease. How it affects the diseases is not just a question of zinc status, but also an issue of mutations in the many proteins that maintain cellular zinc homoeostasis, such as zinc transporters of the ZIP (Zrt-/Irt-like protein) and ZnT families and metallothioneins, and the multiple pathways that change the expression of these proteins. Cadmium interferes with zinc’s functions and there is increased uptake under zinc deficiency. Remarkably, cadmium exposure through inhalation is now recognized in the activation of macrophages to a pro-inflammatory state and suggested as a trigger of a specific form of nodular RA. Here, we discuss how these metal ions participate in the genetic, metabolic, and environmental factors that lead to joint destruction. We conclude that both metal ions should be monitored routinely in arthritic disease and that there is untapped potential for prognosis and treatment.

## 1. Introduction

Among the common rheumatic diseases, osteoarthritis (OA) and rheumatoid arthritis (RA) are thought to be the most prevalent ones, with approximately 11% (10% OA and 1% RA) of the world’s population being affected [1]. According to epidemiological data, people living in industrialised countries are more likely to suffer from these forms of arthritis when compared to developing countries, with Europe and the US leading as these forms are more common in Caucasians [2]. Both conditions affect later stages in life (>45 years), with OA prevalence rising indefinitely with age. With a longer life expectancy and an increase of ageing populations, there is no doubt that arthritis places a huge burden on individuals and society, making it a tremendous public health issue. For example, in 2013, the impact to US economy of arthritis-attributable medical care costs and earning losses in individuals with arthritis was estimated to be $303.5 billion, which was 1% of the US Gross Domestic Product in that year [3].

Although OA and RA are described as two different arthritic conditions (Table 1) with OA being a degenerative disease and RA an autoimmune disease, they share many risk factors and pathogenic characteristics, resulting mainly in cartilage destruction and bone degeneration [4,5]. The pathology of both conditions is well described, yet their aetiology still remains unknown. Increasing age, environmental (smoking), nutritional (diet), and hormonal (female gender) factors, obesity, genetics, and epigenetics seem to trigger the activation of the synovium with release of pro-inflammatory cytokines into the synovial fluid of the joint leading to chronic inflammation (synovitis). As a result, joints are affected by inflammation in both conditions causing swelling, pain, stiffness, and eventually destruction, which in many cases lead to disability.

RA is a heterogeneous disease and as such it has different types and subtypes. The presence of autoantibodies (seropositivity) or their absence (seronegativity) determines the overall type of RA. The first type is associated with formation of immune complexes by rheumatoid factor antibodies (RF), and antibodies against post-translationally modified proteins such anti-citrullinated protein antibodies (ACPAs), and the more recently discovered antibodies against carbamylated [6] and acetylated proteins [7], and manifests with more severe symptoms and joint damage, and increased mortality [8,9]. On the other hand, three different subtypes of RA are mainly determined by the levels of synovial and systemic inflammation (high, low, mixed) based on histology and gene expression profiles [10]. Another distinct subtype only recently postulated is a form of nodular RA, which is characterized by pulmonary rheumatoid nodule formation and generation of RA-associated autoantibodies caused by cadmium inhalation [11].

Apart from cadmium involved in nodular RA, zinc is thought to be involved in arthritis as it has an essential role in the innate and adaptive parts of the immune system, in regulating different aspects of the inflammatory response, and in bone growth and regeneration [12,13,14]. Evaluation of zinc content in the hair of non-smoking and smoking RA patients has shown significantly decreased zinc concentrations and significantly increased cadmium concentrations in the latter [15]. Similarly, the total zinc levels in the sera of RA sufferers were low and negatively correlated with the levels of secreted pro-inflammatory markers [16,17]. Yet, a comprehensive overview of RA from a medical perspective does not mention these metal ions at all [18]. In this review, therefore, we attempt to explain this discrepancy and discuss the involvement of these metals in the pathogenesis of arthritis.

## 2. Zinc and Cadmium in Inflammation and Autoimmunity

Inflammation is the essential response of the innate immune system to a trauma/injury or an infection in order to protect the host from tissue damage or a spread of the infection. Both RA and OA present with an early and late unresolved inflammatory response of the joints (synovitis), respectively, which often results in joint destruction. RA, in contrast to OA, is an autoimmune disease. Growing evidence points at key roles of zinc in the pathogenesis of both conditions as zinc is involved in many inflammatory and immune processes and tissue homoeostasis. Much as zinc deficiency has adverse effects, exposure to cadmium also has adverse effects. Cadmium exerts some of its effects through inhibiting thiol-containing antioxidant enzymes and thus changing the redox balance.

### 2.1. Inflammation

In humans, two classes of zinc transporters, the ZIP (Zrt-/Irt-like proteins, SLC39A) and ZnT (SLC30A) families with a total of 24 proteins, regulate cellular zinc. ZIP transporters pass zinc from the extracellular fluid or from intracellular vesicles into the cytoplasm, while ZnT transporters facilitate movement of zinc out to the extracellular space or sequestration of cytoplasmic zinc into intracellular compartments. A dozen metallothioneins (MTs), small, cysteine-rich, metal-binding proteins, also have a function in controlling cellular zinc, and they bind cadmium tightly [19]. MTs have a critical role in infections, immunity, and inflammation [20]. All these proteins are integrated into the cellular signal transduction network.

Inflammation can trigger an acute phase response that removes zinc (and iron, but not copper) from the plasma. Involved in this reaction is interleukin (IL) 1β produced from macrophages, cortisol produced in the adrenal gland and adrenocorticotropic hormone (ACTH) produced in the pituitary gland. IL-1β, via induction of nitric oxide (NO), induces Zip14 in the liver and leads to sequestration of zinc [21]. Glucocorticoids and several cytokines such as IL-1β, IL-6 in particular, and tumour necrosis factor-α (TNF-α) induce MT [22].

A study addressing the regulation of zinc homoeostasis during OA onset and progression found ZIP8 markedly increased in osteoarthritic human chondrocytes as compared to normal ones, making it an important metal transporter among ZIPs under these conditions [23]. Conversely, suppression of ZIP8 in murine OA chondrocytes protects from cartilage degradation. Yet, proportional addition of zinc to both RA and OA synoviocytes modifies ZIP8 expression, indicating that ZIP8 functions enhance the pathogenesis of RA and OA. These changes in zinc homoeostasis are thought to indicate a resistance of synoviocytes to a build-up of too much zinc, which would lead to their apoptosis. Intriguingly, the metal-regulatory transcription factor 1 (MTF1) seems to be a critical transcription regulator of hypoxia-inducible factor (HIF)-2α, which in turn up-regulates ZIP8, increasing cellular zinc, and MTF1-dependent transcriptional activity [24]. Consequently, the ZIP8-zinc-MTF1 axis and HIF-2α interact in promoting OA cartilage destruction.

MTs, on the other hand, are thought to be biomarkers of the pathogenic status of arthritis. They are elevated in rheumatoid arthritis [25,26] with MT-1 and MT-2 gene expression being associated with more severe autoimmune diseases in viable moth-eaten mice [27]. Furthermore, arthritic models such as collagen-induced arthritis (CIA) and collagen antibody-induced arthritis (CAIA) in mice showed a significant suppression of inflammatory symptoms when MT-1 was expressed intra-articularly. Intraperitoneal MT-1/-2 injections in mice decreased pro-inflammatory mediators and suppressed arthritis through tumour growth factor-β production [28]. However, as RA progresses serum levels of MTs decline, a phenomenon that is reversed by cortisone administration with the result of significantly improved symptoms [29]. Collectively, these observations demonstrate a protective and possibly a therapeutic role of MT in RA.

In both OA and RA, inflammatory cytokines such as IL-1β and TNF-α stimulate the production of matrix metalloproteinases (MMPs), such as MMP-1, -3, -8, -9, -13, and ADAMTS5 [30,31,32,33,34,35]. MMPs are a family of zinc-dependent proteins, which accumulate in the synovium during inflammation and have a central role in cartilage destruction in arthritis [36]. Among the MMPs involved in cartilage degradation, MMP-13 is the primary MMP expressed by chondrocytes and synovial cells in human OA and RA. It cleaves type II, IX, X collagen and other extracellular matrix components (e.g., fibronectin, aggrecan, fibromodulin). In murine OA chondrocytes, inhibition of MMP-13 activity by targeting ZIP8 regulation with miR-488 (microRNA found in chondrocytes) recovers chondrocyte differentiation/cartilage development [37].

### 2.2. Autoimmunity

RA is an autoimmune disease that leads to extra-articular manifestations primarily affecting the lining membrane of the joints, the synovium. The systemic effects of the disease seem to take place at its very onset. To date, no cause has been identified although several possibilities have been suggested. One factor could be a perturbation of zinc metabolism as zinc has critical cellular and molecular functions of zinc in both the innate and adaptive immunity and affects the susceptibility to infections [38]. Indeed, RA is strongly linked with microbial infections [39]. In this article, we discuss whether low serum zinc indicates a systemic zinc deficiency that is involved in the autoimmune aspects and pathogenesis of the disease or it is merely a pathophysiological effect, i.e., a consequence of an acute phase reaction that removes zinc from the circulation. Numerous investigations in isolated immune cells or living organisms provide now insights into how zinc deficiency or supplementation can alter both B-cell and T-cell receptor signaling. In addition to possible implication for diagnosis, whether the immune responses and the inflammation in RA can be influenced with agents that modulate zinc homoeostasis clearly is a critical aspect for management and treatment of the disease.

### 2.3. Innate Immunity

Zinc regulates the function of macrophages and natural killer cells (NK) in many ways. For example, prolonged zinc deficiency diminishes the integrity of lysosomes, activates the nod-like receptor family pyrin domain containing 3 (NLRP3) inflammasome to produce active caspase-1 (interleukin-converting enzyme) and causes the secretion of the pro-inflammatory cytokine IL-1β secretion from macrophages [40]. In monocytes, zinc deficiency reduces the production of IL-6 and TNF-α [41]. IL-1β is an important mediator of inflammation and is involved in a variety of cellular activities, such as cell proliferation, differentiation and apoptosis. IL-6 and TNF-α also are pro-inflammatory cytokines that can be measured in the serum and the arthritic synovial fluid of patients with RA after synovial inflammation develops [42,43], hence playing a significant role in the occurrence and development of RA, more specifically in bone erosion and osteoclastogenesis (formation of bone-resorbing osteoclasts) [44,45]. However, the overall effects of zinc deficiency on the production of IL-6 and TNF-α remain unclear, as the results are inconsistent depending on the compound and the dose of cell-stimulation (e.g., lipopolysaccharide (LPS) vs. phytohaemagglutinin (PHA)) [46,47,48]. On the other hand, zinc modulates endotoxin-induced (LPS) human macrophage inflammation through zinc transporter ZIP8 induction and transcription factor C/EBPβ inhibition [49].

Zinc deficiency also seems to decrease natural killer (NK) cells and thus their cytotoxic effects and the production of IL-2, which is responsible for the activation of T cells and stimulation of NK proliferation. NK cells are responsible for the first line of defense against some pathogens, but through interaction with other cells such as dendritic cells, macrophages, and T cells they contribute to autoimmune diseases such as RA [50]. The binding of nuclear factor kappa-light-chain-enhancer of activated B cells (NF-κB) to DNA is decreased in zinc deficiency [51]. This is of great interest as NF-κB signaling has a critical role in a wide range of cellular activities such as the production of anti-apoptotic factors, cell cycle regulators, cytokines and chemokines, and adhesion molecules and thus NK cell growth and differentiation, maturation of dendritic cells, differentiation of inflammatory T-cells, and polarization of macrophages to the pro-inflammatory state. Therefore, this pathway is an important factor in specific gene expression in response to inflammatory cytokines and oxidative stress in the pathogenesis of OA and RA [52]. Zinc has an anti-inflammatory effect on the NF-κB pathway [53,54].

### 2.4. Adaptive Immunity

Zinc is involved in the maturation of dendritic cells (DCs) in vivo [55]. DCs interface with the adaptive immune system, and during disease they may initiate autoimmune responses and stimulate T cells with resultant macrophage activation to the pro-inflammatory type and ensuing significant tissue damage. Notably, LPS stimulation decreases intracellular “free” zinc in splenic CD11c+ DCs, increases surface expression of major histocompatibility complex (MHC) II molecules, and alters ZIP6 transporter expression in splenic CD11c DCs. On the contrary, zinc supplementation or overexpression of the gene encoding ZIP6 inhibits LPS-induced up-regulation of MHC II and co-stimulatory molecules [56]. The higher expression of MHC II molecules, as also seen in zinc deficiency, is strongly involved in RA susceptibility [57].

Evidence to date also suggests that zinc inhibits a variety of pro-inflammatory responses on T cells and B cells, which confer susceptibility to autoimmune diseases such as RA. An experimental human zinc deficiency caused an imbalance between T helper 1 (Th1) cell and T helper 2 (Th2) cell responses by decreasing the production of interferon-γ (IFN-γ) and IL-2 cytokines, both products of Th1, while not affecting the production of IL-4, IL-6, and IL-10 cytokines, products of Th2 [51]. Th1 cytokines are well known for their pro-inflammatory actions responsible for killing intracellular parasites and for perpetuating autoimmune disorders. IFN-γ up-regulates MHC class I and class II molecule expression. Furthermore, cytoplasmic zinc represses memory T helper 17 (Th17) cell responses in humans by inhibiting the signal of IL-1β. As a result, zinc decreases the production of IL-17 from memory Th17 cells, up-regulated by LPS stimulation, suggesting that zinc availability can affect memory CD4^+^ T cell responses [58]. In arthritis-prone BXD2 mice, IL-17 seems to induce effective B-cell differentiation, thus having a role in the pathogenesis of autoimmune disease [59]. It may explain why B cell development in the bone marrow is still blocked during zinc deficiency by reducing pre-B- and immature B-lymphocytes or by the zinc transporter ZIP10, setting the threshold of B cell receptor signal strength and thus controlling antibody-mediated immune responses [60]. ZIP7 also has a role in B cell receptor signaling. Hypomorphic mutations in ZIP7 result in a phenotype with reduced cytoplasmic “free” zinc and in an autosomal recessive disease with a block of B cell development [61]. In CIA and CAIA models, MT-1 inhibits the differentiation of Th17 cells but favours that of Treg cells [62]. This observation emphasises the role of MT-1 in the disease as discussed in 2.1.

While the effects of induced zinc deficiency on various immune cells is well investigated, low plasma zinc levels (hypozincaemia) can be the result of an acute phase response during autoimmune disorders such as RA. Acute inflammation up-regulates ZIP14 via IL-6 [63], inducing liver sequestration of zinc and redistribution [64]. Moreover, exposure of synovial cells from RA patients to IL-17 and TNF-α increases the expression of zinc exporter ZnT1 and MTs and results in enhanced zinc export and in further increases of inflammation and IL-6 production. These experiments demonstrate a feedback loop between the acute phase inflammatory response and cellular zinc uptake [65].

Of critical importance is the effect that zinc has on T cell development and maturation by binding to thymulin, a nonapeptide hormone secreted by the thymus gland. Thymulin promotes T cell maturation, cytotoxic action, and IL-2 production [66]. The dependence of thymulin on zinc explains some outcomes of zinc deficiency on T cell responses. In both animal and human models, zinc deficiency is associated with thymus gland atrophy and subsequent impaired thymulin activity, which is restored by zinc supplementation. Zinc binding of thymulin and zinc dependence of its activity has been characterized [67]. Furthermore, although it is poorly understood which transporter regulates T cell differentiation in the thymus gland, ZIP3 has an ancillary role in zinc homoeostasis to generate naïve T cell populations in the thymus [68]. There is variation of MT in thymus development, with significant differences noted in New Zealand Black mice, which develop autoimmune diseases [69].

## 3. Zinc and Cadmium in the Aetiology and as Risk Factors of OA and RA

Both OA and RA are multifactorial conditions with many genetic and non-genetic factors involved in their pathogenesis. It is thought that a series of events or triggers from non-genetic factors such as age, gender, trauma, and malalignment of the joint, high body-mass index (BMI), diet, infection, and cigarette smoking in combination with an arthritis-prone genetic profile are responsible for the onset of arthritic disease. Among the factors involved, genetics, gender, and hormones, cigarette smoking, diet, and infection are linked to zinc and cadmium.

### 3.1. Genetics

There is a clear effect of the expression of certain genes in both OA and RA patients when compared to healthy individuals. Advances in technologies over the last few years in both genetics and metallomics combined with large, well-characterized clinical cohorts have allowed us to recognize interactions that are critical in the clinical expression of the diseases.

More than a hundred loci are associated with RA risk, most of which involve immune mechanisms [70]. The human leukocyte antigen (HLA) system, specifically HLA-DRB1 seems to be the most dominant, and it is related to peptide (and self-peptide) binding in pathogenesis [71]. Patients with RA expressing HLA-DRB1 have a higher activity of zinc-dependent MMP-3, an enzyme which degrades cartilage [72]. Among other important genetic loci (e.g., PTPN22, PADI4) with polymorphisms that have smaller functional effects, CD40, a receptor localized on the cell surface of many immune cells including B lymphocytes, contributes to RA pathogenic mechanisms via co-stimulatory pathways. CD40 is linked to zinc transporter ZnT7 expression in the regulation of immune function of human B lymphocytes. Knockdown of ZnT7 in B lymphocytes decreases expression of CD40 on the cell surface [73]. Conversely, the A/A genotype, rs430759, of the zinc transporter ZnT6 is associated with a low susceptibility to RA severity, suggesting a protective role of this particular genotype [74]. Genome-wide association studies looking at genetic susceptibility factors in OA have discovered many important loci with a couple of novel genetic variants being relevant to zinc. Particularly an intergenic variant, rs2820436, of zinc-finger (CCCH-type) containing 11B pseudogene (ZC3H11B) is associated with OA across any joint, whereas another variant, rs375575359, resides in the zinc finger protein 345 gene (ZNF345) and is strongly associated with knee OA [75]. These investigations highlight the value of new approaches to address the metabolism of essential metals, such as zinc, in the genetics of arthritic disease with the prospect of early detection and better management.

### 3.2. Gender

Although the aetiology of RA and OA remains unclear, some pathogenesis patterns are well-known, with the female gender being one of them. Women have a higher tendency to develop several autoimmune diseases and are affected by RA 2.6 times more than men [76], with age of menarche, oral contraceptive use, termination of pregnancy, lactation, and menopause playing important roles [77]. Similarly, women are more likely to suffer from OA, particularly in the years after menopause [78]. Hormonal alterations, with oestrogen reduction being discussed most frequently, in those women are therefore confounding factors. Oestrogen inhibits the production of T helper cell products such as IL-1β, TNF-α, which can activate MMPs for cartilage destruction [79]. Oestrogen also induces anti-inflammatory effector functions in immunoglobulin G (IgG) by inducing β-galactoside α-2,6-sialyltransferase (1St6Gal1) expression in antibody-producing cells and by increasing immunoglobulin Fc fragment sialylation in mice and in patients with RA [80]. Zinc deficiency can alter androgen and oestrogen receptor levels while it impairs the synthesis/secretion of follicle-stimulating hormone (FSH) and luteinizing hormone (LH) [81]. On the other hand, zinc supplementation in ovariectomized rats increases serum oestrogen and progesterone levels [82]. The exact mode of action of zinc on these hormonal systems is unknown. However, an established molecular link is that the nuclear receptors for sex steroids contain zinc finger motifs in their protein structure. Conversely, administration of hormones such as oral contraceptives, which contain progesterone and oestrogen, seems to increase serum copper and ceruloplasmin levels in humans and rats, while hepatic zinc stores are elevated [83]. In a large cross-sectional study of the Korean National Health and Nutritional Examination Survey, RA in females was associated with increased cadmium in sera [84]. Cadmium can be an endocrine disruptor and considered a “metallo-oestrogen” because of its oestrogenic actions.

Mast cells (MCs) serving as a cellular link among numerous components (FcγRIII, IL-1, PKC/NF-κB) in inflammatory arthritis increase both in RA patients and in animal models [85]. The fact that mast cell-associated diseases, including allergy/anaphylaxis and neuroinflammatory pain disorders, exhibit a gender bias towards women could also explain the high prevalence of RA in females [86].

### 3.3. Cigarette Smoking and Occupational Exposure

Cigarette smoking is associated with the development of both RA and OA [87,88], with men being at a greater risk. In contrast to OA where no exact mechanism has been described, autoimmune components of RA are induction of citrullination of peptide antigens and rheumatoid nodules present in the lungs that also cause the production of ACPAs. In smokers with RA, ACPAs have been mainly seen in patients carrying the HLA-DRB1 gene. A single cigarette contains as much as 3 µg of cadmium, which has a half-life of 15–20 years in the human body and is a type 1 carcinogen [89,90,91]. Next to diet, cadmium in tobacco is the second most important factor for cadmium exposure.

Cadmium exposure in the lungs of laboratory animals has emerged as an important process by which antigen presenting cells generate and present citrullinated proteins/peptides [92]. Given that the process is induced in vivo by cadmium and that cadmium levels are higher in smokers [93,94] the association of cadmium inhalation from smoking with nodule formation in the lungs of RA patients is the strongest evidence yet for a causative involvement of metal ions in the disease [11]. In addition to smoking, epidemiological evidence for the association between cadmium inhalation and nodular rheumatoid arthritis stems from occupational settings such as workers in the mining, steel, textile, and other industries [95,96,97]. It is thought that the inhaled cadmium leads to nodules (granulomas) in the lung and the observed formation of RF, ACPAs and other autoantibodies that are typical of RA. Rheumatoid arthritis-associated interstitial lung disease and other lung diseases can predate the development of rheumatoid arthritis by many years before symptoms in the joints develop. Pulmonary disease also can develop later. It is one of the extra-articular manifestations of RA and a major factor in mortality [98,99]. Therefore, understanding the molecular basis of how cadmium initiates RA will provide further insights into the role of metal ions in the disease. A possible pathway could be through the expression of ZIP8 observed in the lung of chronic smokers compared to controls. Considering the three facts that (i) cadmium is similar to zinc and accordingly the biological actions of cadmium(II) ions have been described by some as ionic mimicry with regard to zinc(II) ions, (ii) ZIP8 can be a portal for cadmium entry into cells, and iii) ZIP8 expression in lungs depends on the NF-κB pathway, it is contended that ZIP8 plays a significant role in cadmium toxicity, and the subsequent effects on zinc metabolism, in the smoking-induced lung disease [100].

### 3.4. Infection

Both animal and human studies support the pathogenic role of infection in RA. The strongest evidence exists for *Porphyromonas gingivalis (P. gingivalis)*, a bacterium frequently found in periodontitis. The proposed mechanism is via endogenous expression of peptidylarginine deiminase (PADI), an enzyme essential for the generation of citrullinated autoantigens [101]. Other infectious agents such as *Proteus mirabilis*, *Escherichia coli*, and Epstein-Barr virus are thought to trigger RA, but none of these claims have been substantiated further [39].

The intersection between risk groups of RA and zinc deficiency in serum is often described. Considering that zinc regulates the proliferation, differentiation, maturation and functioning of lymphocytes, and other leukocytes [102], zinc deficiency will increase susceptibility to infectious agents, including *P. gingivalis*. On the other hand, in vitro, zinc ions markedly enhance the adhesion and accumulation of salivary and serum proteins on cells of *P. gingivalis* while they inhibit its co-aggregation and haemagglutination [103]. Moreover, an arginine solution including zinc and dentifrice attenuates the deleterious effects of *P. gingivalis* on keratinocyte barrier function and its ability to translocate through a gingival epithelium model [104]. Zinc oxide nanoparticles (ZnO NPs) in the infected root canal are antibacterial at low concentrations and with low cytotoxicity [105]. Zinc can therefore limit the settlement of *P. gingivalis* in the gingival sulcus, preventing periodontal disease and subsequent development of RA. Examination of the direct effect of zinc supplementation on *P. gingivalis* infection is indicated.

The intestinal microbiota has also been implicated in inducing arthritis in some people, an effect that seems to be modulated by the individual’s genetic background. Patients with early RA have an intestinal microflora with a high prevalence of different arthritogenic bacteria such as *Prevotella copri*, *Lactobacillus salivarius*, and *Collinsella* [106]. Because the host microbiota competes with the host for zinc, zinc deficiency—as seen in the sera of RA patients—may be related to preferential growth of those bacteria that are able to survive at low zinc levels. In contrast, high dietary zinc reduced the expression of enterobacteria with increasing age in weaning piglets, but permanently changed Lactobacillus species. The high zinc diet, however, did not affect other species such as Bifidobacteria, Enterococci, Streptococci, *Weissella* spp., and *Leuconostoc* spp. as well as the *Bacteroides*-*Prevotella*-*Porphyromonas* group [107]. Indeed, use of zinc increases the presence of Gram-negative facultative anaerobic bacterial groups, the colonic concentration of short chain fatty acids (SCFAs), as well as overall species richness and diversity [108]. Hence, zinc supplementation could be a preventive strategy in RA patients, making novel nutritional interventions, including prebiotics and probiotics regulating the gut microbiota, worth investigating with regard to RA.

## 4. Metabolism

Zinc deficiency re-programs the immune system with immediate effects on infection but also longer-term effects [109]. Among longer-term consequences are thymic atrophy, lymphopenia, and compromised innate and adaptive immunity that lead to a higher incidence and a longer duration of infections. Sufficient zinc is anti-inflammatory and many molecular pathways in which zinc is involved in immunity have been mapped [38]. Inflammation can change metabolism, an effect referred to as metabolic re-wiring or re-programming of the immune system. TCA (Krebs) cycle metabolites and derivatives have non-metabolic, signaling functions in the immune system [110,111]. At least three metabolites of the TCA and urea cycles have been discussed in RA: itaconic acid, succinic acid, and citrulline. Itaconate and succinate are now recognized as having important signaling roles [112], which are critical for inflammation control by macrophages.

### 4.1. Itaconate (Methylene Succinic Acid)

Itaconate (IA) is a product of the TCA cycle and an immunomodulatory compound involved in immune tolerance. Activated macrophages synthesize it because the mitochondrial enzyme immune-responsive gene-1 (IRG1) is induced and produces IA from the decarboxylation of cis-aconitate [113]. Nitric oxide (NO) has a central role in this induction [114]. IA inhibits succinate dehydrogenase (SDH) with the consequence of less FADH_2_ and fumarate being formed. It leads to a metabolic remodelling of macrophages and controls inflammation [115]. IA links innate immune tolerance and adaptive immunity and can be anti-inflammatory through its action on two transcription factors [116,117]. It alkylates Keap1 (Kelch-like ECH-associated protein 1), which activates Nrf-2 (Nuclear factor erythroid 2-related factor 2), and suppresses IL-1β production [118]. It also modifies the thiol group of glutathione forming an adduct that stimulates ATF3 (Activating Transcription Factor 3) (in addition to Nrf-2), which suppresses IL-6 production [119]. IA is involved in the post-translational modification (PTM) of other proteins, a process that is reversed by the enzyme SIRT5 (sirtuin 5). IA is found in immune cells but its secretion is low and therefore it is not a biomarker of systemic inflammation [120].

### 4.2. Succinate

IA inhibits SDH and accordingly increases succinate, which can be detected in the synovial fluid of RA patients [121]. In addition to its effect on mitochondrial respiration succinate can be exported and then extracellular succinate participates in controlling inflammation by binding to a G-protein coupled receptor (GPR91, SUCNR1) and modulating HIF-1α responses. Thus, succinate exacerbates RA through the GPR91 pathway [122]. Succinate is also involved in PTM of lysine side chains.

### 4.3. L-Citrulline

Citrulline is a product of the ornithine transcarbamoylase reaction in the urea cycle. It is also formed via nitric oxide synthase, which synthesizes NO and L-citrulline from L-arginine and oxygen with the aid of the cofactor tetrahydrobiopterin. The enzyme is a dimer with an intersubunit zinc site. Another reaction produces citrullinated proteins directly. It is catalysed by peptidylarginine deiminase (PADI4), which takes off ammonia from the side chain of arginine in a Ca^2+^-dependent reaction. A polymorphism of PADI4 is associated with RA. In RA, there is increased formation of citrullinated proteins and autoantibodies are formed against these proteins as one of the major aspects of it being an autoimmune disease. Another modification concerns the side chain of lysine in proteins. In a reaction involving myeloperoxidase the cyanate produced from thiocyanate carbamylates the lysine to form homocitrulline [123].

Metabolomics/metabonomics is used to follow metabolic changes in RA and to develop biomarkers. Among the 20 metabolites found in synovial fluid from RA patients were succinate and citrulline and others from the urea and TCA cycles and fatty acid and amino acid metabolism [124]. A more recent investigation found at least 30 metabolites as potential biomarkers [125].

### 4.4. Role of Zinc and Cadmium in Metabolic Re-Programming

In addition to being a catalytic and structural cofactor in over 3000 human enzymes, zinc(II) ions are considered signaling ions [126]. Zinc is not directly involved as a catalytic metal in the enzymes of either the TCA cycle or the urea cycle but it is thought to have regulatory functions. For example, mitochondrial aconitase is zinc-inhibited [127,128] and human ornithine transcarbamoylase has a putative zinc inhibition site [129]. Thus, changes in the availability of zinc will affect the fluxes through these cycles. Furthermore, there is control of arginine levels through the zinc inhibition of dimethylargininase-1 [130]. Another form of metabolic signaling is the isoprenoid-dependent PTM of proteins (“prenylation”) involved in differentiation or maintenance of effector Treg cells, which are important for immune tolerance [131]. The enzymes involved in the farnesylation and geranylgeranylation are zinc enzymes [132,133].

Cadmium is ten times better absorbed through inhalation in the lung than from the diet in the intestine, and therefore lung inflammation and disease is a major concern for exposure. Macrophages have a dual role. They are involved in inflammation and repair. Cadmium exposure in the lung leads to metabolic re-programming of macrophages with a switch to glycolysis and the pro-inflammatory phenotype [134]. In this process, mitochondrial reactive oxygen species (ROS) increase and affect redox-sensitive transcription factors. Even low levels of cadmium in drinking water affect energy metabolism in the lung [135]. Because the ZIP8 and ZIP14 zinc transporters also transport cadmium, exposure to cadmium exacerbates the effects of zinc deficiency on the innate immune system and the pro-oxidant effects of zinc deficiency in inflammation [136]. These effects include a polymorphism in ZIP8 (Ala391Thr) that is associated with inflammatory disease and bacterial infection.

## 5. Oxidative Stress

RA is said to be a disease with oxidative stress, a term that is now divided into eustress for physiological oxidative stress and distress for pathological oxidative stress [137]. A detailed discussion of the oxidative damage in RA has been provided [138]. Because reactive species are also necessary for redox signaling, the debate about whether antioxidants are good or bad for the treatment of RA remains largely unresolved. Redox metabolism intersects with metal metabolism, and even redox-inert metal ions such as zinc and cadmium exert a significant control over redox metabolism in an indirect way. Both zinc deficiency and overload generate redox stress. A major issue is the production of NO in macrophages, which has been linked to the metabolic re-programming and the production of IA. NO reacts with the sulfur donor in zinc/thiolate coordination environments and releases zinc [139,140]. Similar observations have been made for cadmium [141]. NO is also involved in the activation of MMPs [142]. A cysteine-switch mechanism activates the zymogen: The cysteine when bound to zinc keeps the enzyme inactive, but the oxidative modification of the thiolate ligand of cysteine leads to dissociation of the cysteine and activation of the enzymes [143]. Thus, there is a clear connection between metal metabolism, reactive species, and intermediary metabolism.

When blood parameters indicative of oxidative stress were investigated in 120 patients with RA and compared to 53 healthy controls, an increase of ROS, lipid peroxidation, protein oxidation, DNA damage and a decrease in antioxidant defense was noted in the RA patients [144]. When 29 RA patients and 41 healthy controls were compared, the RA patients had high antioxidant levels in their blood, but they were not sufficient to avoid oxidative damage [145].

Two relatively recent articles provide a literature review on the topic of oxidative stress in RA. A systematic review in 2016 confirmed the involvement of oxidative stress in the disease. It did not make definite conclusions, however, due to controversies from literature data between 2010 and 2015 [146]. An article in 2019 formulated a consensus from a limited number of clinical trials that adjuvant therapy with antioxidants has a potentially beneficial effect [147]. No specific biomarkers for the disease were suggested, though.

Nrf-2 k.o. mice have significantly more tissue damage in antibody-induced arthritis than control mice [148], indicating that oxidative stress is involved in cartilage destruction. The authors also reported Nrf-2 activation in the joints of patients.

Other than NO and the role of nitric oxide synthase, there is virtually no specific information about which other reactive species are involved in RA and what their targets are.

## 6. Clinical Applications

A substantial number of patients adequately respond to current therapies for arthritis. Yet there are patients whose response remains inadequate because of therapy’s poor efficacy and lack of specificity. The primary aim of the treatments remains to decrease pain and inflammation associated with disease activity, but many of the regimens still fail to effectively prevent cartilage damage and further progression of the disease. Moreover, due to the fact that many treatments lose their efficacy over time, there is the need to increase the dose which in turn augments their toxicity and side effects. To date, the first line for RA treatment includes immunosuppressant disease-modifying anti-rheumatic drugs (DMARDs), with methotrexate being the dominant one, and glucocorticoids. The next class of remedies is biological agents (bDMARDs), a group of different classes of monoclonal antibodies affecting the body’s response to various cytokines, while non-steroidal anti-inflammatory drugs (NSAIDs) and corticoid intra-articular joint injections are used for treating both RA and OA.

The increasing need for an improvement in the treatment for synovial hyperplasia and subsequent joint destruction, as seen in both late RA and OA, has not been overcome by current therapies and has led to the investigation of other therapeutic parameters. Metal-related therapies (gold, zinc, selenium, iron, copper, manganese, and cadmium) show encouraging results due to their involvement in inflammatory, immune and oxidation processes; however, neither one of these regimens has a role in the traditional therapy nor are they approved as drugs in the US.

### 6.1. Zinc Interventions for OA and RA Therapy

The different therapeutic interventions with zinc in both OA and RA are summarized (Table 2). Zinc homoeostasis affects chondrocyte matrix synthesis and proliferation in animal and in vitro studies, with the level of response being relevant to the source of the chondrocytes (growth plate vs. articular cartilage), developmental stage (fetal, newborn, or adult), as well as the culture methods used. It is now well established that variation of zinc levels is associated with pathological changes in cartilage. In fact, intracellular zinc levels and ZIP8 expression are elevated in human OA chondrocytes, which in turn increase the expression of multiple proteolytic enzymes (MMP-3, -9, -12, -13, and ADAMTS5) capable of promoting OA. Conversely, when ZIP8 was conditionally knocked out in chondrocytes there was a reduction of cartilage destruction in mice [149]. A similar reduction in articular cartilage damage of the osteoarthritic knee joint of rats (monoiodoacetate model, MIA) was observed after receiving oral zinc [150]. Moreover, a nutraceutical formula containing zinc (Phytalgic^®^) used in a small placebo-controlled study on OA (knee or hip) showed a remarkable improvement in symptoms (Western Ontario and McMaster Universities Osteoarthritis Index—WOMAC), suggesting a therapeutic benefit of zinc in OA [151]. A significant reduction in the inflammatory marker C-reactive protein (CRP) and in assessment criteria (Disease Activity Score, DAS-28) was also noticed with the use of low-dose zinc in conjunction with antioxidants such as selenium, and vitamins C/A/E (Selenplus, Eurovital^®^) in women suffering from RA (mild-to-moderate severity) in an Iranian pre-post clinical trial [152]. This observation indicates the beneficial role of zinc, and other micronutrients, in alleviating the oxidative stress seen in RA (discussed earlier).

Although studies looking at the effects of zinc supplementation in RA show a reduction in patients’ monocytes (in vitro) and an increased phagocytosis of blood polymorphonuclear cells (PMNs) [153,154], two important immune processes for amelioration of symptoms, they seem to be contradictory regarding the overall clinical effect. The first researcher to indicate a link between RA and zinc was Peter A. Simkin, who in 1977 reported improvements in joint swelling, morning stiffness and walking in RA patients whose diet was supplemented with zinc [155]. Later studies failed to produce any beneficial effect measurable with the selected biomarkers, even though the zinc supplement provided was three times the US RDA [156,157]. Nevertheless, zinc treatments were suggested [158]. RA patients were found to have significantly lower urinary excretion of zinc 24 h after its ingestion compared to the control group, suggesting zinc malabsorption or deficiency [159]. In a prospective study of 29,000 women in the US, with 152 RA cases identified over 11 years, greater use of supplemental zinc was inversely associated with RA [160].

Specific zinc-dependent MMPs have been targeted to treat RA, yet the compounds failed to show efficacy due to their lack of selectivity and the many adverse effects [161]. Advances in biology and drug design, though, have yielded novel agents with desired selectivity, which might be successful for the therapy of RA [162]. Of greatest interest is the inhibitor of MMP-14 referred to as membrane type 1 MMP (MT1-MMP) which together with bDMARDs causes the reduction of cartilage collagen degradation in the mouse CIA model [163]. The inhibition of MMP-9 in fibroblast-like synoviocytes (FLSs) by andecaliximab, a monoclonal antibody, resulted in the promotion of survival, invasion, and the release of pro-inflammatory cytokines by FLSs, yet it proved to be unsafe [164]. These studies suggest that, although it is unlikely for MMPs to be the sole target for treatment of RA, targeting MMPs may increase the efficacy of other drugs, especially in the context of intra-articular delivery. On the contrary, cleavage of zinc-dependent MMP-13 and ADAM/ADAMTSs has been recently pursued as potential targets for OA [165]. In particular, MMP-13 inhibitors PF152 and the less toxic ALS 1–0635 and 43a (PF152 successor) have displayed chondroprotection in human articular cartilage in vitro and in vivo animal models of OA [166,167,168]. Finally, ADAM17 and ADAMTS5 may alter the physiological and mechanical properties of the extracellular matrix and hence the progression of arthritis [169,170].

### 6.2. Cadmium Interventions for OA and RA Therapy?

The use of low dose cadmium intra-articular injection in the adjuvant induced arthritis (AIA) rat joints can control inflammation, preserve juxta-articular bone, and prevent cartilage destruction [23]. Apparently, increased cadmium concentrations in synoviocytes are related to reduced cell viability, cell proliferation and IL-6 levels (in supernatants), with OA synoviocytes showing higher sensitivity to cadmium than the RA ones. Furthermore, ZIP8 and MT-1 expression, usually increased in arthritis, seem to be negatively associated with cadmium. Although ZIP8 is known to be a major portal for cadmium entry in cells and so is its homologue ZIP14 [171], cadmium transporters and MT expression and their response to the inflammatory and cadmium stimuli in chondrocytes and bone cells have not yet been identified. Though the levels of acute administration of cadmium via an injection remain subtoxic—corresponding to exposing humans to the smoke of three cigarettes [23]—the classification of cadmium as a class I carcinogen would seem to prevent its use as a therapeutic agent for local OA pathogenesis. Identification of the sites of action of cadmium could identify additional disease mechanisms involving the essential metal ions with which this toxic metal ion interferes [89].

## 7. Conclusions

The biological functions of redox-inert zinc and cadmium, the congener of zinc in the same group of the periodic table, are distinctively different from the transition metals (manganese, iron, copper), which are redox-active in biology, and from magnesium and calcium, which are also redox-inert but bind to biomolecules with lower affinity and do not interact with the sulfur ligand of cysteine. It is known for at least 50 years that patients with RA have low zinc in their sera. Accordingly, treatment with zinc supplements was suggested but met with mixed results. The situation is similar to the uncertainties surrounding the efficacy of antioxidants in treating the oxidative stress in arthritic joint disease. The reason for these uncertainties is to be found in the tight regulation of nutritionally essential zinc in tissues where slight changes from homoeostatic control in the physiological range can have either pro-antioxidant or pro-oxidant effects [172]. Changes in the availability of cellular zinc ions elicit potent effects on many pathways in the immune system, inflammation, and in cartilage and bone metabolism. Some of these effects have been mapped to signaling zinc ions as ligands in the modulation of protein tyrosine phosphatase activity [173]. It remains unclear whether patients with inflammatory arthritis, which is an on-going process without immediate, if any, resolution of the inflammation, have altered zinc status in the affected tissues and possibly are zinc deficient. New insights into the many proteins that control zinc metabolism and mutations in these proteins emphasise the importance of gene-micronutrient interactions in arthritic diseases. Cadmium is present in our diet, tobacco smoke, and dust, and some workers are exposed to it in specific occupational settings. Very recent insights suggest that this non-essential and toxic metal ion has a role in the aetiology of some forms of nodular RA. We posit that the metallobiochemistry underlying zinc and cadmium provides new insights into arthritic diseases. While these metal ions have practically no place in the traditional treatment and in the description of arthritic diseases in academic medicine, restoration of perturbations of zinc metabolism, and control of cadmium intoxication provide significant opportunities for treating and diagnosing these devastating diseases. One major advance will be the analysis of the metal status in tissues. While our discussion focused on OA and RA, the other three major arthritic diseases, fibromyalgia, lupus erythematosus and gout, and the less common ones, also should receive some scrutiny with regard to the principles discussed here.

## Figures and Tables

**Table 1:** Differences between osteoarthritis and rheumatoid arthritis.

	Osteoarthritis	Rheumatoid Arthritis
Onset	Progressive, >40 years of age	Rapid, 20–50 years of age
Type of disease	Degenerative (wear and tear)	Autoimmune
Types of joints	Overused or weight-bearing joints, Uni- or Bilateral	Small joints of hands and feet, but may involve large joints and spine, Symmetrical/Bilateral
Morning stiffness	<20 min	>1 h
Pathophysiology	Loss of cartilage, Subchondral hypertrophy, cysts and sclerosis, Osteophytes present; Decrease of joint space	Inflammation of synovial membrane (extra-articular manifestations), Osteophytes absent, Joint destruction
Systemic symptoms	−	Anaemia, Fatigue, Weight loss, Osteopenia
Common Blood Bio-markers	Normal ESR * Normal to slightly elevated CRP ** Absence of RF Absence of ACPAs	Normal to elevated ESR * Normal to elevated CRP ** Presence or absence of RF Presence or absence of ACPAs

* Erythrocyte Sedimentation Rate; ** C-Reactive Protein.

**Table 2:** Therapeutic interventions with zinc in osteoarthritis and rheumatoid arthritis.

Disease	Effect (*p*-Value)	Treatment	Study Method	Reference
OA	Increased subchondral plate thickness, reduction of cartilage damage (*p* < 0.001)	Knockout of ZIP8	in vivo (Chondrocyte-specific CKO fl/fl mice (Mtf1; Col2a1-Cre))	Kim, J.H.; et al. [149]
OA	Increased serum antioxidative capacity, glutathione levels, and IL-10 levels; lower Osteo-arthritis Research Society International (OARSI) scores (*p* < 0.05) Reduced IL-1β and MMP-13 expression; increased ROS production (*p* < 0.05)	1.6 mg/kg/day of zinc supplementation; 25 μM zinc	in vivo (*n* = 5, mono-sodium-induced iodoacetate (MIA) Wistar rats) in vitro (chondrosarcoma cell line (SW1353 cells) treated with 5 μM MIA)	Huang, T.C.; et al. [150]
OA	Decreased cartilage degeneration (cartilage breakdown products TIINE, ARGN, and AGEG) (*p* < 0.001)	MMP-13 inhibitor PF152	ex vivo (human cartilage explants) in vivo (n = 60, beagle dogs with OA-induced by partial medial meniscectomy)	Settle, S.; et al. [165]
OA	Chondroprotection	MMP-13 inhibitor 43a	in vivo (n = 2 male Sprague-Dawley IGS rats) (n = 5 male beagle dogs) (n = 2 cynomolgus monkeys)	Ruminski, P.G.; [166]
OA	Reduction of cartilage damage (*p* < 0.05)	MMP-13 inhibitor ALS 10635	in vivo (n = 12, MIA-induced OA male Sprague-Dawley rat) (n = 20 surgical induced OA male Lewis rats)	Baragi, V.M.; et al. [167]
OA	Inhibition of aggrecanase activity (*p* < 0.001)	Humanized anti-ADAMTS-5 monoclonal antibody, GSK2394002	in vivo (*n* = 20 DMM male Swiss Webster mice)	Larkin, J.; et al. [169]
OA	Blockage of IL-6R	ADAM17, ADAM inhibitor, GW280264X	in vitro (cultured cells)	Ludwig, A.; et al. [170]
OA	Lower use of analgesics (*p* < 0.001) and /or NSAIDs (*p* < 0.02), improved WOMAC scale (*p* < 0.001)	*Phytalgic* capsules three times daily: 10 mg zinc citrate, 1371 mg fish oil, 60 mg nettle leaves Urtica dioica L., 12 mg Vitamin E, 12 mg Vitamin C	Double-blind randomised Controlled Trial (*n* = 81)	Jacquet, A.; et al. [151]
RA	DAS-28 score and serum hs-CRP (*p* < 0.01); increased antioxidant markers (TAC, GPX, SOD, and CAT) (*p* < 0.01)	One *Selenplus* capsule daily: 50 μg selenium, 8 mg zinc oxide, 400 μg vitamin A, 125 mg vitamin C, and 40 mg vitamin E	Pre-post Controlled Trial (*n* = 40 female)	Jalili, M.; et al. [152]
RA	No beneficial effect on inflammation or clinical indices	220 mg zinc sulphate (45 mg elemental zinc) three times daily	Controlled Trial (*n* = 18)	Peretz, A.; et al. [153]
RA	No considerable alterations in ROS amounts of monocytes	130 mg zinc aspartate two times daily	in vivo and in vitro Following treatment with zinc, after in vitro monocyte stimulation with 0.1 mM zinc (II)	Herold, A.; et al. [154]
RA	positive changes regarding joint swelling, morning stiffness, walking time; no improvements regarding grip strength	220 mg zinc sulfate (45 mg elemental zinc) three times daily	Double-blind Controlled Trial (*n* = 24)	Simkin, P.A. [155]
RA	Increased phagocytic activity of PMNs; unknown clinical effect (lack of statistical analysis)	45 mg elemental zinc daily (zinc gluconate)	Controlled Trial (*n* = 22)	Peretz, A.; et al. [156]
RA	No change in ESR, HCT or joint scores in severe RA patients (lack of statistical analysis)	220 mg zinc sulfate (45 mg elemental zinc) three times daily	Controlled Trial (*n* = 22)	Rasker, J.J.; et al. [157]
RA	No statistically significant therapeutic effect; increase of alkaline phosphatase (*p* < 0.01)	220 mg zinc sulfate (45 mg elemental zinc) three times daily	Double-blind Randomised Controlled Trial (*n* = 27)	Mattingly, P.C.; et al. [159]
RA	Greater use of supplemental zinc (*p*-trend = 0.03) was inversely associated with rheumatoid arthritis	Supplemental zinc (10.4-15.5 mg/day) or food only containing zinc (9.7-13.5 mg/day)	Prospective cohort study (*n* = 29,368 females)	Cerhan, J.R.; et al. [160]
RA	Reduced cartilage degradation and disease progression (*p* < 0.05)	MT1MMP selective inhibitory antibody DX2400 and/or TNFRFc fusion protein	in vivo (CIA-treated mice)	Kaneko, K.; et al. [163]
RA and OA	Inhibition of MMP-9; promotion of survival, invasion and release of pro-inflammatory cytokines by FLS (*p* < 0.01)	Andecaliximab	in vitro (OA and RA synovial fibroblasts)	Xue, M.; et al. [164]

## Data Availability

Not applicable.

## Abbreviations

1St6Gal1	β-galactoside α-2,6-sialyltransferase
ACPAs	anti-citrullinated protein antibodies
ACTH	adrenocorticotropic hormone
AIA	adjuvant induced arthritis
bDMARDs	biologic disease-modifying anti-rheumatic drugs
BMI	body-mass index
CAIA	collagen antibody-induced arthritis
CIA	collagen-induced arthritis
CRP	C-reactive protein
DC	dendritic cells
DMARDs	disease-modifying anti-rheumatic drugs
ESR	erythrocyte sedimentation rate
FLSs	fibroblast-like synoviocytes
GDP	gross domestic product
HIF-2α	hypoxia-inducible factor-2α
HLA	human leukocyte antigen
IA	itaconate (methylene succinic acid)
IFN-γ	interferon-γ
IgG	immunoglobulin G
IL	interleukin
IRG1	immune-responsive gene-1
LPS	lipopolysaccharide
MC	mast cells
MHC	major histocompatibility complex
MIA	monoiodoacetate model
MMPs	matrix metalloproteinases
MT	metallothionein
MTF1	metal-regulatory transcription factor 1;
NF-κB	nuclear factor kappa-light-chain-enhancer of activated B cells
NK	natural killer cells
NLRP3	nod-like receptor family pyrin domain containing 3
NO	nitric oxide
OA	osteoarthritis
PADI4	peptidylarginine deiminase
PHA	phytohaemagglutinin
PMNs	blood polymorphonuclear cells
PTM	post-translational modification
RA	rheumatoid arthritis
RF	rheumatoid factor
ROS	reactive oxygen species
SCFAs	short chain fatty acids
SDH	succinate dehydrogenase
SOD	superoxide dismutase
TCA	Krebs cycle (tricarboxylic acid cycle)
Th1	T helper 1 cells
Th17	T helper 17 cells
Th2	T helper 2 cells
TNF-α	tumour necrosis factor-α
ZIP	Zrt-/Irt-like protein
ZnO NPs	zinc oxide nanoparticles
